# Serum insulin concentration in dogs with insulinoma as a clinical marker for presence of metastasis at the time of diagnosis

**DOI:** 10.1111/jvim.16720

**Published:** 2023-05-16

**Authors:** Andrea Petrelli, Alexander J. German, Erin M. O'Connell, Paolo Silvestrini

**Affiliations:** ^1^ Institute of Veterinary Science University of Liverpool Neston UK; ^2^ School of Veterinary Medicine University of Pennsylvania Pennsylvania Philadelphia USA

**Keywords:** insulinemia, prognosis, stage, survival

## Abstract

**Background:**

Information regarding serum insulin concentration in dogs newly diagnosed with insulinoma and its association with clinical stage and survival time is lacking.

**Objective:**

Examine association between serum insulin concentration and survival and clinical disease stage in dogs with insulinoma.

**Animals.:**

Fifty‐nine client‐owned dogs with a diagnosis of insulinoma from 2 referral hospitals.

**Method:**

Retrospective observational study. The *χ* ^2^ test was used to compare the proportion of dogs with increased insulin concentration in groups with or without metastasis at the time of diagnosis. Linear mixed‐effect models were built to compare differences in insulin concentration between dogs with and without evidence of metastasis at the time of original diagnosis. Cox's proportional hazards regression and Kaplan‐Meier graphs were used to evaluate the association between insulin concentration and insulin groups and survival.

**Results:**

Median serum insulin concentration was 33 mIU/L (range, 8‐200 mIU/L) in dogs with World Health Organization (WHO) stage I disease and 45 mIU/L (range, 12‐213 mIU/L) in dogs with WHO stage II and III disease. No difference was found in the proportion of dogs with increased insulin concentration with or without metastasis (*P* = .09). No association was identified between insulin concentration and survival (*P* = .63), and between dogs grouped by insulin concentration and survival (*P* = .51).

**Conclusions and Clinical Importance:**

Serum insulin concentrations were not different between dogs with or without metastasis at diagnosis. The degree of insulinemia does not provide further information regarding the stage of the disease and is not associated with survival time in dogs with insulinoma.

**Abbreviations**
ACTH: adrenocorticotropic hormone
BIC: Bayesian information criterion
CT: computer tomography
EBVS: European Board Veterinary Specialization
IRMA: immunoradiometric assay
RIA: radioimmunoassay
WHO: World Health Organization

## 1 INTRODUCTION

Insulinoma is a functional tumor of pancreatic beta cells, characterized by insulin secretion independent of blood glucose concentration.^1^ Insulinoma has been reported in different species including humans,^2^, ^3^ dogs,^4^ and cats.^5^, ^6^, ^7^ Many different clinical signs are observed, including seizures, collapse, generalized weakness, muscle twitching or trembling, ataxia, exercise intolerance, pelvic limb weakness, and disorientation.^8^, ^9^, ^10^ These signs are thought to be the result of either neuroglycopenia or hypoglycemia‐induced catecholamine secretion.^11^, ^12^, ^13^

In human medicine, as many as 90% of insulinomas are reported to be benign whereas, in veterinary medicine, most dogs have microscopic or macroscopic metastasis at the time of diagnosis.^1^, ^6^ The most common sites of metastasis are locoregional lymph nodes, liver, and lungs; metastasis to the spleen, mesentery, gastrointestinal tract, kidney, spinal cord, and bone has been described but are much less common.^1^, ^4^, ^8^, ^9^, ^13^, ^14^ Clinical staging of insulinomas according to the WHO defines stage I as T_1_N_0_M_0_ (primary tumor with no evidence of metastasis), stage II as T_1_N_1_M_0_ (primary tumor with locoregional lymph node metastasis) and stage III as T_1_N_1_M_1_ or T_1_N_0_M_1_ (primary tumor with distant metastasis with or without locoregional lymph node metastasis).^15^ Treatment of choice is surgical resection of the primary mass and lymph nodes with metastases if present.^4^, ^8^, ^14^ For nonsurgical candidates, medical treatment can be considered. This treatment includes attempts to decrease insulin secretion (e.g., diazoxide, octreotide), to increase insulin resistance and promote gluconeogenesis (e.g., glucocorticoids) or cytotoxic treatment (e.g., streptozocin, tyrosine kinase inhibitors).^1^, ^4^, ^8^, ^16^, ^17^, ^18^, ^19^, ^20^

Detection of metastases at the time of diagnosis is fundamental to select the most appropriate treatment approach. Their presence may influence cost of treatment, treatment success rate, and prognosis. Unfortunately, despite advanced diagnostic techniques such as contrast‐enhanced computed tomography (CT), abdominal ultrasonography, and fine needle aspiration of suspicious intra‐abdominal lesions, metastases can be missed. In addition, economic constraints of clients and the inability for some veterinary facilities to perform such procedures should be considered. Therefore, development of a simple inexpensive method to predict the presence of metastasis at time of diagnosis would be useful in the management of these cases. Two recent studies have described clinically relevant differences between benign and malignant insulinomas in people.^21^, ^22^ Compared to benign tumors, malignant insulinomas tended to be larger, be associated with higher plasma insulin concentrations and to be associated with metastases.^21^, ^22^ However, it is not known whether the same is true for insulinomas in dogs. Therefore, our primary aim was to compare serum insulin concentration in dogs with insulinoma and WHO stage I disease to those with WHO stage II or III disease and to establish if an association existed between stage and other variables and serum insulin concentrations at the time of diagnosis. Our hypothesis was that serum insulin concentration would be higher in dogs with metastatic insulinoma compared to dogs with stage I disease and that the degree of insulinemia would be correlated with survival.

## 2 MATERIALS AND METHODS

### 2.1 Study design and ethics

Ours was a retrospective observational study examining serum insulin concentrations in dogs with insulinoma. Institutional ethical approval for retrospective data acquisition was obtained before the start of the study (Veterinary Ethics Review Committee [VERC] of the University of Liverpool, approval number 1002) and all owners gave written consent.

### 2.2 Case identification and eligibility criteria

A search of electronic clinical records of dogs that attended 2 veterinary referral hospitals between January 2013 and January 2021 was conducted. To be eligible for inclusion, each dog required a diagnosis of insulinoma, based on presence of hypoglycemia (defined as a blood glucose concentration below the reference interval used by both institutions [65‐130 mg/dL], either at diagnosis or after a period of fasting) with concurrent inappropriate serum insulin concentration (either within or above the reference interval). Blood glucose concentration was measured on whole blood using a portable glucose meter (AlphaTrak) or on serum obtained by centrifugation within 15 minutes after collection of a blood sample in serum gel tube using a biochemical analyzer (Beckman Coulter AU480), both methods validated in dogs.^23^, ^24^ Serum insulin concentration was measured either by radioimmunoassay (RIA) or immunoradiometric assay (IRMA). The RIA has been validated in dogs,^25^ whereas the IRMA was internally validated against the RIA by the laboratory (NationWide Laboratories, UK) using approved procedures which included parallelism, intra‐assay and inter‐assay variation as well as limit of detection and recovery studies using samples from dogs. The same IRMA assay was used in a recent study measuring insulinemia in dogs undergoing surgical management of insulinoma.^14^ The same serum sample in which hypoglycemia was identified was used to quantify insulinemia, once other causes of low blood glucose concentration were excluded. If the sample volume was not sufficient or had not been maintained at or below 4°C, a new blood sample was obtained and, if hypoglycemia was confirmed, the sample was placed in a refrigerator pack and sent to the respective external laboratory for insulin measurement. In all cases, serum insulin concentration was assessed using the same sample in which hypoglycemia was documented.

Evidence of a pancreatic nodule on abdominal imaging (CT or ultrasonography) also was required for inclusion. In addition, thoracic CT or 3‐view thoracic radiography also was required to stage each dog using the TNM system. Finally, data needed to be available from history, physical examination, hematology, serum biochemistry and, if available, either the cytological or histopathological report of the pancreatic nodule and of the suspected metastatic lesion. The diagnosis of insulinoma still was considered likely even in dogs without cytological or histopathological examination of the pancreatic mass, based on compatible clinical signs, normal or increased serum insulin concentration with concurrent hypoglycemia, and presence of a pancreatic mass on CT or ultrasonography. Dogs with relapsed disease and those in which staging had either not been performed or had been performed ≥30 days after serum insulin measurement were not eligible. Dogs were also ineligible if medications had been administered that could have influenced serum glucose or insulin concentrations (e.g., glucocorticoids). Most other causes of hypoglycemia including sepsis, liver failure, and extrapancreatic neoplasia were excluded based on history and physical examination and routine hematology, serum biochemistry, and full body imaging. Basal serum cortisol concentration was assessed in dogs suspected of hypoadrenocorticism.

### 2.3 Data collection and grouping

The electronic client data management systems of the 2 referral hospitals were searched for dogs with a diagnosis of insulinoma. Data gathered from medical records included signalment (age, sex, neuter status, and breed), clinical signs at the time of presentation, results of clinicopathological investigations (including CBC, serum biochemistry, blood glucose concentration, and serum insulin concentration), and time of death. Preoperative diagnostic imaging findings, including identification of a pancreatic nodule or mass, presence or absence of metastasis on radiography, ultrasonography, or CT were retrieved from diagnostic imaging reports conducted by or overseen by either a European Board of Veterinary Specialization (EBVS) European Veterinary Specialist in Diagnostic Imaging or an American Veterinary Specialist in Radiology. Diagnostic imaging procedures either were performed at the first appointment or soon after receiving the serum insulin concentration results. Ultrasound‐guided fine‐needle aspiration of a pancreatic nodule or of potential metastasis was performed at the end of the imaging procedure (CT or ultrasonography) and cytology samples were evaluated by either an EBVS European or American Veterinary Specialist in Clinical Pathology. Surgical reports were reviewed and compared with the preoperative imaging findings. Histopathologic examination was performed on the excised primary pancreatic nodule or mass and, when available, on suspected metastatic tissue (lymph node or otherwise) either by an EBVS European or American Veterinary Specialist in Pathology. Dogs were grouped according to the WHO staging system (stage I, T_1_N_0_M_0_; stage II, T_1_N_1_M_0_; stage III, T_1_N_1_M_1_ or T_1_N_0_M_1_) into those with stage I disease (Group A) and those with either stage II or III disease (Group B). Serum insulin concentrations were classified as normal (within the reference interval) or high (insulin concentration greater than the upper limit of the reference interval).

### 2.4 Statistical analysis

Data were entered into an electronic spreadsheet (Microsoft Excel for Mac version 16.19) and checked for errors, and an online open‐access statistical language and environment (R, version 4.2.0)^26^ was used for all statistical manipulations, and the level of statistical significance was set at *P* < .05 for 2‐sided analyses. A *χ* ^2^ test was used to compare the proportion of dogs with increased serum insulin concentration in groups with or without metastasis at the time of diagnosis, and the sensitivity and specificity of having an increased serum insulin concentration above the reference interval also was determined.

Given that different insulin assays were used at the different veterinary hospitals, linear mixed‐effects models were built (using the “lme4” package version 1.1‐29^27^) to compare differences in insulin concentration between dogs with and without evidence of metastasis at the time of original diagnosis. For these analyses, insulin assay was included as a random effect and other variables included as fixed effects. Initially, a series of simple models was created consisting of a single fixed effect in addition to the random effect (insulin assay), with variables tested including age, sex, neuter status, duration of clinical signs before diagnosis, presence of clinical signs (weakness, seizures, collapse, ataxia, appetite change) and WHO stage (stage I vs stage II or III). A multiple mixed‐effects regression model then was built which initially included any of the variables that were *P* < .2 when included as the sole fixed effect in simple regression. The model then was refined by backwards stepwise elimination of variables, with the Bayesian information criterion (BIC) being used to select the model with the best generalizability. Models were validated by checking normality of residuals using the Shapiro‐Wilk test and for asymmetry in distribution by calculating skewness and visual inspection of a histogram plot. If assumptions were not met, datasets were logarithmically‐transformed and the analyses repeated. Cox's proportional hazard regression (Cox PH regression) was performed to evaluate the associations between survival and either serum insulin concentration or insulin group (normal vs higher than reference interval). For these analyses, survival time was calculated as the time from the diagnosis until the death of the animal (either by euthanasia or natural causes). For the serum insulin concentration, the model was stratified based on the insulin reference range (because different methods had been used at different referral hospitals). Kaplan‐Meier curves also were used to compare survival in dogs with either normal or increased serum insulin concentration.

## 3 RESULTS

### 3.1 Animals

Sixty dogs with a diagnosis of insulinoma were identified from the 2 referral hospitals. However, 1 dog was excluded because of a pre‐existing diagnosis of hyperadrenocorticism. Therefore, 59 dogs ultimately were included in the study (Table 1) with 32 male dogs (54%; 26 neutered, 6 intact) and 27 female dogs (46%; 25 spayed, 2 intact). Median age at diagnosis was 9 years (range, 4‐14 years). Breeds included crossbreed (11; 19%), West Highland white terrier (7; 12%), Jack Russell terrier (5; 8%), Beagle (3; 5%), 2 each of English springer spaniel, Boxer, Staffordshire bull terrier, Labrador retriever, Chihuahua, and Yorkshire terrier, and 1 each of 21 other breeds.

**TABLE 1:** Breed distribution according to the institution of origin.

Variable	Institution 1 UK	Institution 2 USA
Cases	30	29
Age (month)	114	108
Sex	12MN 4 M 1F 13FN	14MN 2 M 1F 12FN
Breed	4 JRT	8 Cross Breed
	4 WHWT	3 Beagle
	4 Cross Breed	3 WHWT
	2 ESP	2 Chihuahua
	2 Boxer	2 YST
	2 SBT	Basset Hound
	Great Dane	German Shepherd dog
	Kelpie	Labrador Retriever
	Border Collie	JRS
	Irish Setter	Boston Terrier
	Hungarian Visla	Pomeranian
	Flat Coated Retriever	CKCS
	German Wire Haired Pointer	Dalmatian
	German Shorthaired Pointer	Cane Corso
	Shetland Sheepdog	Bernese Mountain Dog
	Labrador Retriever	English Bulldog
	Yugoslavian Sheep Dog	
	Greyhound	
Blood glucose (mg/dL)	45	41

Abbreviations: CKCS, Cavalier King Charles Spaniel; ESP, English Springer Spaniel; JRT, Jack Russel Terrier; SBT, Staffordshire terrier; WHWT, West Highlands White Terrier; YST, Yorkshire Terrier.

### 3.2 Clinical signs

Median duration of clinical signs before diagnosis was 30 days (range, 0‐240 days). Reported clinical signs included generalized or focal seizures (24 dogs; 40%), weakness (22 dogs; 37%), ataxia (8 dogs; 33%), collapse (5 dogs; 17%), and lethargy (5 dogs; 17%).

### 3.3 Laboratory testing and staging

Median blood glucose concentration at presentation was 43 mg/dL (range, 16‐88 mg/dL). The dog with normoglycemia at presentation (88 mg/dL) became hypoglycemic (54 mg/dL) after a short period of fasting. Median serum insulin concentration was 48 mIU/L (range, 8‐213 mIU/L).

Basal serum cortisol concentration was measured in 16 (27%) dogs and an adrenocorticotropic hormone (ACTH) stimulation test was performed in 7 (12%). All of these dogs had normal basal or post‐ACTH serum cortisol concentration, making hypoadrenocorticism an unlikely cause of hypoglycemia. Pre‐prandial bile acid concentrations were measured in 9 (15%) dogs and a bile acid stimulation test was performed in 5 (8%). Four (28%) of these dogs had a minimal increase in pre‐prandial or post‐prandial bile acid concentration that was not considered clinically relevant.

All dogs were staged by thoracic and abdominal imaging. Thoracic and abdominal dual‐phase contrast CT was performed in 42 dogs (71%), in 16 (27%) dogs a combination of thoracic and abdominal radiography and abdominal ultrasonography was performed, and in 1 dog thoracic radiographs and abdominal ultrasonography were followed by thoracic and abdominal CT. Group A included 27 dogs (46%) and Group B 32 dogs (54%). In Group B, 21 dogs (35%) were classified as WHO stage II and 11 (19%) as WHO stage III. Fine needle aspiration of the pancreatic nodule or mass and extrapancreatic lesions if present was performed in 9 dogs (15%). Histology after partial pancreatectomy was performed in 33 dogs (56%). All cytological and histological samples were consistent with a neuroendocrine tumor and all of the excised nodules and masses that were submitted for histology were classified as carcinomas. Seventeen cases (28%) did not have cytological or histopathological evaluation of the pancreatic mass or the metastasis.

Median serum insulin concentration was 33 mIU/L (range, 8‐200 mIU/L) in dogs of group A, with 12/27 (44%) having concentrations above the respective reference interval. Median serum insulin concentration was 45 mIU/L (range, 12‐213 mIU/L) in dogs of group B, with 19/32 (59%) having concentrations above the respective reference interval. No significant difference was found in the proportion of dogs with increased serum insulin concentrations with or without metastasis (*P* = .09). Sensitivity and specificity of increased serum insulin concentration (above the reference interval) for detecting the presence of metastasis were 0.56 and 0.86, respectively.

**FIGURE 1:**
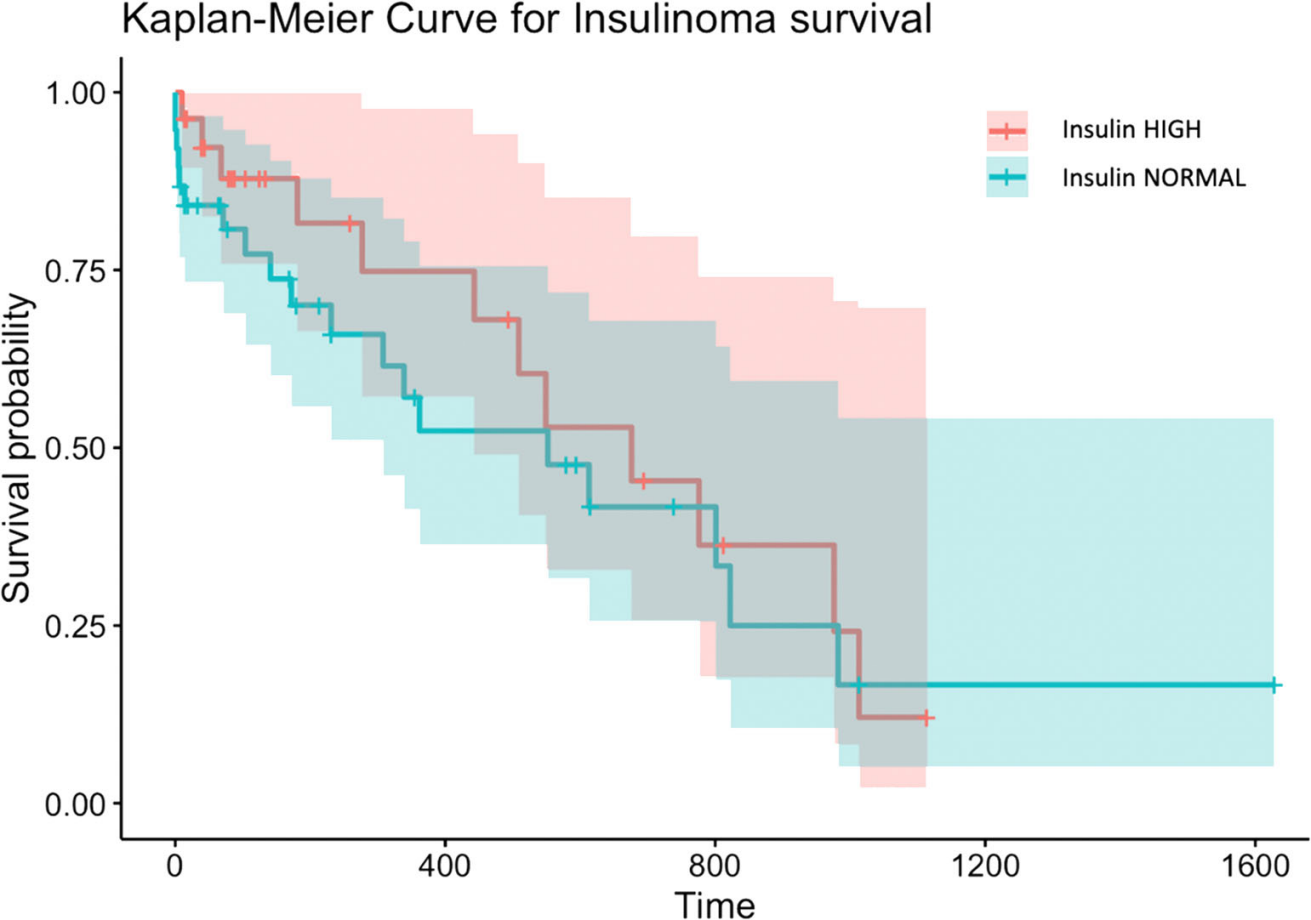
Kaplan‐Meier curve for survival of dogs classified according to insulin level as high or normal.

Using mixed effects models with log insulin concentration as the outcome variable and insulin assay as a random explanatory variable, the only variable of significance was duration of clinical signs (*P* = .03), and presenting with collapse was the only other variable that met the threshold for inclusion (*P* = .13). No association was found between serum insulin concentration and having metastases present at diagnosis, either when included as a single fixed effect in a linear mixed effects model (*P* = .26) or with both duration of clinical signs and presence of collapse as other fixed effects (*P* = .41). No association was found between survival and serum insulin concentration either when assessed as an absolute concentration (Cox PH regression, *P* = .63) or classified as normal vs increased (Cox PH regression, *P* = .51; Figure 1).

## 4 DISCUSSION

Our study is the first to assess serum insulin concentration in dogs with insulinoma in relation to clinical staging. Previous studies demonstrated an association between the presence of metastasis at time of diagnosis and survival time, highlighting the importance of staging dogs with insulinoma.^14^, ^28^, ^29^ This consideration is particularly important because insulinoma in dogs is more often malignant, compared to insulinoma in human patients. Two studies in human medicine investigated the differences between benign and malignant insulinomas. One study reported that, when patients were presented within 24 hours of symptoms appearing, the fasting time for the occurrence of hypoglycemia was <8 hours, the serum insulin concentration was ≥28 mU/mL and C‐peptide concentration was ≥4 ng/mL at the glycemic nadir, malignant insulinoma was more likely.^21^ A tumor >2.5 cm in diameter also was more frequently malignant in people.^21^ Another study confirmed these results and also determined that median insulin (21.0 vs 12.3 μIU/mL), median proinsulin (190 vs 83.5 pmol/L), and median C‐peptide (4.1 vs 3 ng/mL) concentrations were higher in people with malignant insulinomas than in those with benign tumors. This study also confirmed that malignant tumors were larger than benign tumors (4.2 vs 1.8 cm).^22^ The criteria used in human medicine to differentiate a benign vs malignant insulinoma rely on the presence of metastasis, but histological and immunohistochemical analysis is sometimes necessary in those patients without extrapancreatic disease.^30^ In contrast, for dogs included in our study, no difference in serum insulin concentration was found between those with or without metastasis at diagnosis, nor could serum insulin concentrations distinguish benign from malignant primary tumors. Therefore, in dogs, serum insulin concentration at the time of diagnosis has limited prognostic value. The discrepancy between studies in human medicine and our study could be explained by the fact that, in studies of humans, insulin concentration was compared between cases of benign and malignant insulinomas whereas, in our study, all dogs had malignant neoplasia, albeit of different stages. A previous study hypothesized that increased insulin and C‐peptide concentrations might be associated with a larger‐sized primary mass rather than the presence of metastasis,^22^ implying that the amount of insulin produced by metastases might be negligible and thus explaining the lack of difference in insulin concentration in dogs with different disease stages seen in our study. That said, an association between hypoglycemia and clinical signs has been reported in dogs with stage II and III disease, making control more difficult and leading to worse prognosis.^14^ Furthermore, to our knowledge, no previous studies have examined associations between the size of the primary pancreatic mass and serum insulin concentration, regardless of the stage of disease. The size of the primary pancreatic mass was not recorded in many dogs in our study and, therefore, it was not possible to evaluate this aspect. However, no association was found between tumor size and prognosis in a previous study.^14^ Finally, the average duration of clinical signs in the population of our study was 4 weeks, which is consistent with recent studies reporting 4 to 6 weeks since the onset of clinical signs.^10^, ^14^ Our study showed an association between duration of clinical signs and survival, indicating that the longer the duration of the clinical signs, the shorter the survival time. This observation is in contrast with previous studies,^10^, ^14^ in which no significant differences were found between these 2 variables. However, dogs having clinical signs for a longer period of time either because of delayed diagnosis or delayed intervention or both, potentially have more time to develop metastasis, which is associated with worse prognosis.^10^, ^14^ In addition, although the association between the size of the pancreatic mass and a poor prognosis has been demonstrated only in human patients,^21^ with time the insulinoma can become larger with consequently higher insulin production. Prolonged hyperinsulinemic hypoglycemia could become more difficult to control medically and also could induce more severe pancreatic β‐cell dysfunction, with higher risk of postoperative diabetes mellitus.

Our small observational study had some limitations. First, timing of sampling for insulin concentrations was not recorded. In both healthy humans and dogs, insulin secretion follows a circadian rhythm,^31^ which might be disrupted by the presence of insulinoma. Such effects were not considered in our study. Second, given the small population and lack of sample size calculation, it is possible that the study was underpowered, with the potential for type I statistical error. Third, the retrospective and multicenter nature of the study might have introduced variability as a result of differences in diagnostic and therapeutic approach. Most notable was the use of 2 different insulin assays, although an attempt was made to correct for this concern using mixed‐effects and stratification in regression. Fourth, some cases were staged only based on thoracic and abdominal radiographs, wherein sensitivity for metastasis is relatively poor.^32^ Abdominal ultrasonography and conventional CT have sensitivity of approximately 28% to 75% and 71%, respectively, for the detection of a primary mass. Sensitivity can be increased up to 96% using contrast‐enhanced CT.^33^ However, primary masses still can be missed, and surgical exploration might be necessary to confirm them and localize their presence by direct palpation of the pancreas, as noted in a previous study.^14^ Furthermore, contrast‐enhanced CT has a reported sensitivity for detecting lymph node and hepatic metastasis of approximately 67% and 75%, respectively.^33^ This aspect of our study could have caused incorrect classification of some cases. In addition, not all dogs underwent a complete diagnostic evaluation for hypoglycemia. However, the inappropriate insulinemia in relation to the hypoglycemia and presence of a pancreatic nodule or mass made most other causes of hypoglycemia unlikely. Finally, some dogs were included in our study despite no cytological or histological confirmation of insulinoma being performed. One case report described a dog affected by nesidioblastosis,^34^ a condition caused by the hyperplasia of the β‐cells and no detectable pancreatic nodule or mass, and diagnosis was reached only after histological evaluation of pancreatic surgical biopsy samples. In our cases, all dogs without cytological or histological assessment had evidence of a pancreatic nodule or mass.

In conclusion, serum insulin concentration was not associated with either the stage of disease or survival in dogs with insulinoma. These findings confirm and reinforce the importance of clinical staging in dogs with insulinoma in order to provide a more accurate prognosis.

## CONFLICT OF INTEREST DECLARATION

Alexander J. German is an employee of the University of Liverpool, but his post is financially supported by Royal Canin, which is owned by Mars Petcare. Alexander J. German has also received financial remuneration for providing educational material, speaking at conferences, and consultancy work for Mars Petcare; all such remuneration has been for projects unrelated to the work reported in this manuscript. The other authors do not have a conflict of interest to declare.

## OFF‐LABEL ANTIMICROBIAL DECLARATION

Authors declare no off‐label use of antimicrobials.

## INSTITUTIONAL ANIMAL CARE AND USE COMMITTEE (IACUC) OR OTHER APPROVAL DECLARATION

Approved by the Veterinary Ethics Review Committee (VERC) of the University of Liverpool, approval number 1002.

## HUMAN ETHICS APPROVAL DECLARATION

Authors declare human ethics approval was not needed for this study.
